# Nonsurgical Management of Simultaneous Double Lateral Root Perforations in Adjacent Teeth Using CBCT and MTA: A Case Report

**DOI:** 10.1155/crid/2700505

**Published:** 2025-11-04

**Authors:** Beyhan Başkan, Hatice Kübra Başkan, Beyza Güler

**Affiliations:** ^1^Department of Endodontics, Faculty of Dentistry, Kahramanmaras Sutcu Imam University, Onikisubat, Kahramanmaras, Türkiye; ^2^Department of Pediatric Dentistry, Faculty of Dentistry, Kahramanmaras Sutcu Imam University, Onikisubat, Kahramanmaras, Türkiye

**Keywords:** cone beam computed tomography (CBCT), endodontics, iatrogenic perforation, lateral root perforation, mineral trioxide aggregate (MTA)

## Abstract

Lateral root perforations are serious iatrogenic complications that can compromise the outcome of endodontic treatment. The current case report presents the successful treatment of simultaneous double lateral root perforations in the mandibular canine and first premolar using a nonsurgical approach guided by cone beam computed tomography (CBCT) and mineral trioxide aggregate (MTA). Perforations that were not detectable with periapical radiographs were confirmed using CBCT, and the treatment planning was performed based on the millimetric measurements provided by CBCT. The treatment protocol included rubber dam isolation, microscopic assistance, effective irrigation, calcium hydroxide intracanal medication, and definitive sealing of the perforation sites with MTA. The 1-year follow-up revealed asymptomatic teeth with normal function and radiographic evidence of periapical healing. This case highlights that CBCT-guided MTA repair provides a predictable, minimally invasive solution for managing iatrogenic lateral root perforations, though its success is contingent upon a meticulous approach and regular follow-up.

## 1. Introduction

The American Association of Endodontists (AAE) defines a perforation as a mechanical or pathological communication between the root canal system and external tooth surface [1]. This iatrogenic communication compromises tooth integrity and risks periodontal tissue damage [2]. Perforations typically occur during access cavity preparation, canal shaping, or postplacement [3].

Root perforations are serious complications [4]. Untreated cases risk periodontal deterioration and tooth loss, with lateral root perforations posing the greatest threat. Effective, timely repair is essential for tooth preservation [5].

Radiographic detection of labial/buccal or lingual/palatal perforations is often impractical due to superimposition over sound root structure. Electronic apex locators aid in diagnosis, while cone beam computed tomography (CBCT) provides precise three-dimensional details for accurate assessment [6].

Lateral root perforations threaten periodontal health. Nonsurgical repair (preferred for infection control) involves perforation cleaning, debridement, and MTA sealing. MTA's biocompatibility and sealing properties optimize success [7].

MTA, widely used for perforation repair since 1993, offers exceptional sealing and biocompatibility [8]. Its ability to set in moist environments (e.g., blood/water) and calcium silicate–derived osteoconductivity make it ideal for perforations. Studies confirm high success rates with MTA-based repairs [6, 8–11].

Successful root perforation management depends critically on perforation size/location, repair timing, microbial contamination, and material selection—all determining treatment prognosis [12]. While numerous case reports detail the management of single lateral root perforations [9], the simultaneous occurrence and successful nonsurgical repair of double lateral root perforations in adjacent mandibular teeth (canine and first premolar) represent a clinically significant and less frequently documented scenario, increasing the complexity of diagnosis, treatment planning, and execution.

This case report details the process of locating the lateral root perforation in the mandibular canine and first premolar (FDI 33 and 34) using CBCT and treating it with MTA. The patient's history, clinical findings, treatment process, and outcomes are presented in comparison with the existing literature. In this context, the effectiveness of MTA in perforation repair will be highlighted, and the challenges and success factors of the treatment process will also be addressed.

For the treatment and publication purposes presented in this case report, the patient has been informed about all possible risks and benefits, and her written consent has been obtained.

## 2. Case Report

This case report has been written according to the Preferred Reporting Items for Case reports in Endodontics (PRICE) 2020 guidelines [13].

**Figure 1:**
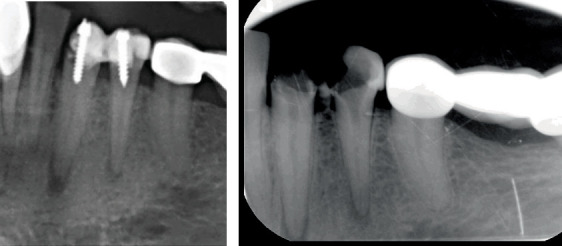
Pretreatment periapical radiograph showing (b) apical radiolucencies in Teeth 33 and 34, along with a (a) metal postcore.

**Figure 2:**
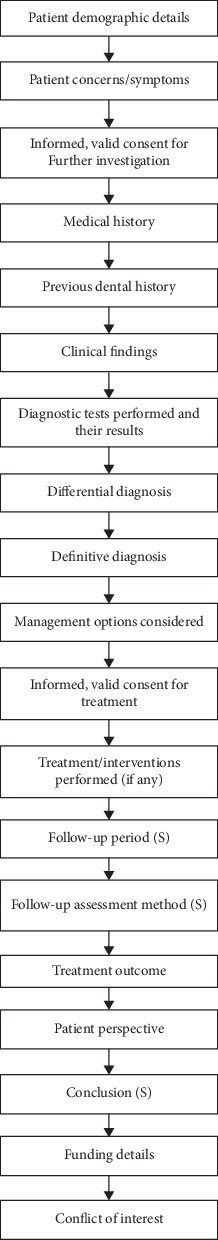
Schematic of the diagnosis and treatment process.

A 41-year-old female patient of Turkish descent was referred to the Endodontics Clinic at Kahramanmaras Sutcu Imam University Faculty of Dentistry due to pain in the left mandibular region. The patient's family history reveals no significant disease conditions. There are no factors in the patient's psychosocial history that could affect the treatment. Genetic testing was not deemed necessary for this case. No pathological findings were observed during the extraoral examination. The patient had no systemic disorders and was not on regular medication. The medical history revealed that approximately 1 year ago, root canal treatment was initiated for Teeth 33 and 34 at a private clinic; however, the treatment was incomplete, and a postcore had been placed. Clinical examination showed percussion sensitivity in Teeth 33 and 34, but no swelling or fistula was observed. Thermal tests did not elicit a vital response. Periapical radiographs revealed radiolucent areas in the apical region of both teeth, the presence of a metal postcore, and unclear canal anatomy (Figure 1). Based on these findings, a diagnosis of asymptomatic periapical periodontitis was made for the teeth. The diagnosis and treatment process are summarized in Figure 2.

**Figure 3:**
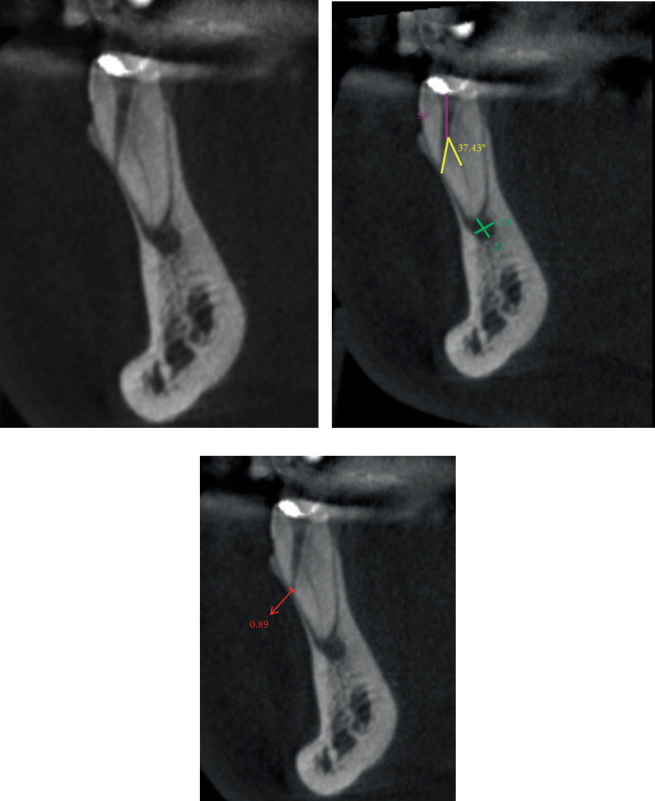
(a) Sagittal section CBCT image of a mandibular canine showing a mesiobuccal perforation. The apex of the tooth was not prepared. (b) In the sagittal section, the distance from the canal mouth to the perforation was 5.8 mm. The angle between the optimal entry vector and the unprepared root canal was 37.43°. Apical lesion measured 2.4 × 2.7 mm. (c) A lateral perforation of 0.89 mm in the apical distobuccal position.

**Figure 4:**
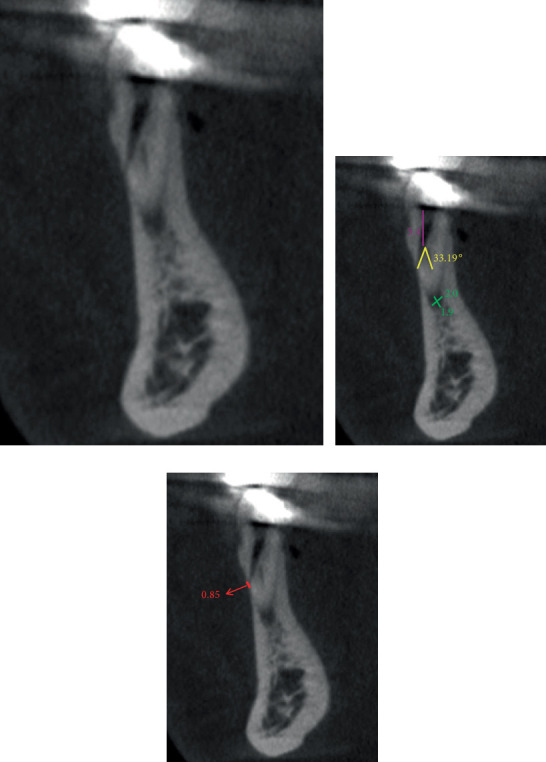
(a) CBCT image in sagittal section showing the mesiobuccal perforation in the tooth. An unprepared root canal was observed at the apex of Tooth Number 34. (b) In the sagittal section, the distance from the canal mouth to the perforation was 5.4 mm. The angle between the optimal entry vector and the unprepared root canal was 33.19°. Apical lesion measured 1.9 × 2.0 mm. (c) A lateral perforation of 0.85 mm in the apical distobuccal position.

While preparing the routine endodontic access cavity under rubber dam isolation, unexpected findings such as persistent bleeding from the canal system and inability to obtain consistent measurements with the electronic apex locator were encountered. This raised suspicion of iatrogenic lateral root perforation. To confirm the diagnosis, CBCT imaging was performed. CBCT findings of Teeth 33 and 34 were recorded (Figures 3 and 4).

**Figure 5:**
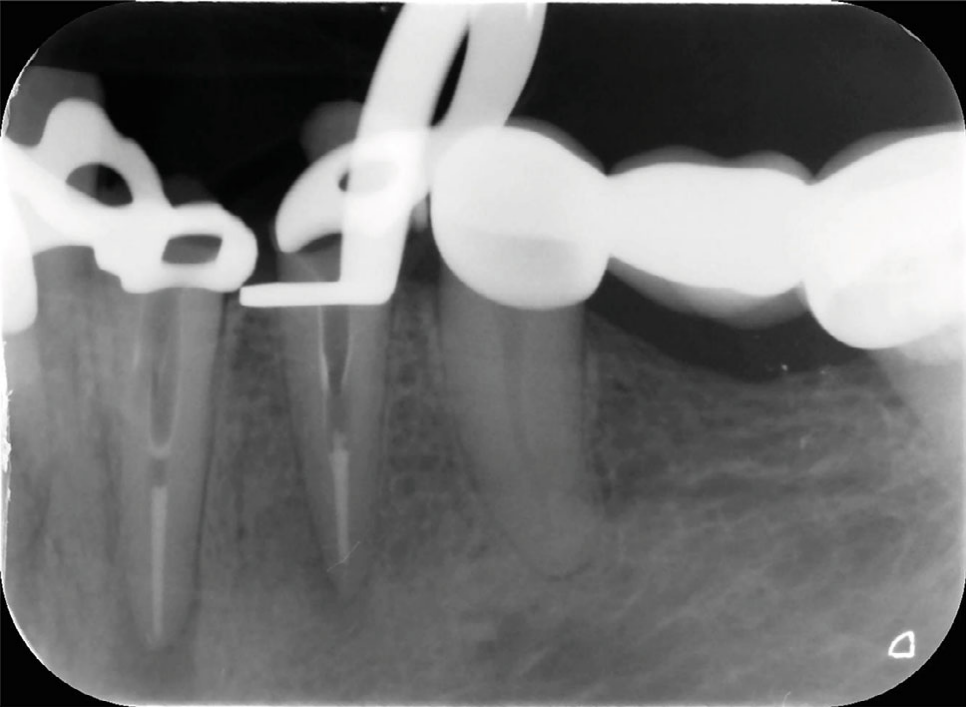
Obturating the apical segments of the root canal using the cold vertical condensation technique.

**Figure 6:**
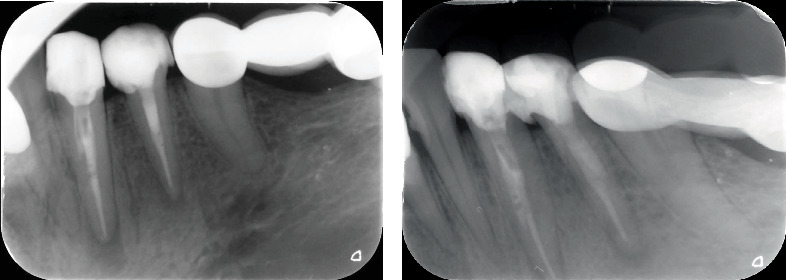
(a, b) Periapical images of Teeth 33 and 34 taken from two different angles; MTA, fiber post, and resin composite applied.

**Figure 7:**
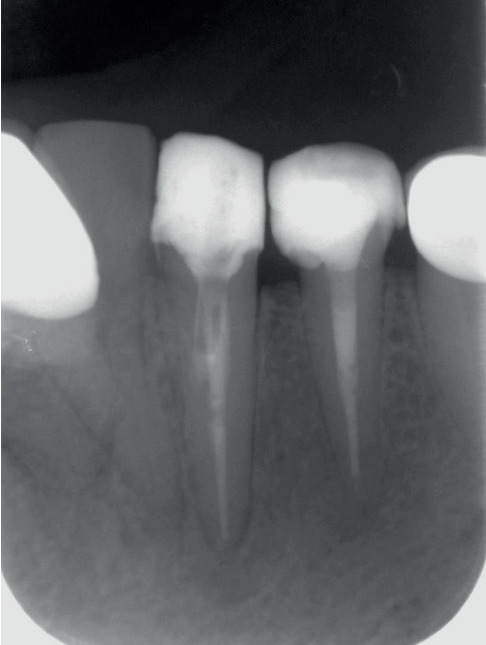
One-year follow-up images of Teeth 33 and 34.

In the treatment, a correct entry angle was obtained based on the CBCT data, and direct access to the unprepared root canals was achieved using a Gates Glidden drill under the magnification of a Zumax Microscope. The canals were irrigated with 2.5% NaOCl (20 mL/tooth) and 17% EDTA (5 mL/tooth) followed by sonic activation. After dressing with calcium hydroxide for 14 days, the root canal segments apical to the perforation were filled with gutta-percha and MTA Fillapex using the cold vertical condensation technique (Figure 5). ProRoot MTA was placed in the perforation sites under microscopic guidance, mixed by hand, and applied using the manual filling technique. To allow for the MTA's setting, damp cotton pellets were left in the canals for 48 h. In the restorative phase, a fiber post and Tokuyama Estelite composite resin were used to create a core (Figure 6). The patient was advised to restore the teeth with a complete crown. Upon 1-year follow-up, the teeth were asymptomatic, the function was normal, and a regression of the apical radiolucencies was observed radiographically (Figure 7).

During the treatment process, porosity containing air bubbles was observed in the MTA perforation area of Tooth Number 33. However, it was noted that this did not have an adverse effect on treatment success after 1 year of follow-up.

## 3. Discussion

Lateral perforation diagnosis is clinically challenging with periapical radiographs [14]. CBCT shows 100% sensitivity versus 50% for periapical radiography [15]. In the present case, perforations that could not be detected with periapical radiographs were successfully confirmed using CBCT. This situation highlights the critical role of three-dimensional imaging in providing nontraumatic access through millimetric measurements, especially in calcified canals.

CBCT imaging was not merely confirmatory but fundamentally altered the treatment strategy. The precise millimetric localization of each perforation (5.8 mm from canal orifice in 33 and 5.4 mm in 34) and, critically, the measurement of the divergent angles between the optimal entry vector and the unprepared canal (37.43° for Tooth Number 33 and 33.19° for Tooth Number 34) enabled the calculation of a nontraumatic, targeted access path using Gates Glidden drills under microscopic guidance. This precise vectoring, unattainable with periapical radiographs, was essential for locating the true canals without exacerbating the perforations [14]. This approach was particularly critical for managing simultaneous double perforations, where precision directly impacted both teeth's prognosis.

In their study, Tuzcu and Kurnaz examined the detection of artificial perforations in teeth using an apex locator and concluded that they were unable to measure perforations of 0.50 mm or smaller [16]. In our current case, an apex locator was used to detect root perforations, and it successfully identified the perforation. Since the diameters of the lateral root perforations were 0.89 mm for Tooth Number 33 and 0.85 mm for Tooth Number 34, the results are consistent with the findings of Tuzcu and Kurnaz. If we were to start treatment without verifying the perforation diameter with CBCT, the likelihood of obtaining false-negative results would increase, potentially resulting in the loss of the tooth due to incorrect treatment.

The literature indicates that rubber dam isolation creates a barrier between the oral environment and the working area, improving the field of vision, while magnification equipment like microscopes provides controlled access [17]. In the current case, controlled access to the root canals was achieved using a microscope and rubber dam isolation.

In their study, Ghasemi et al. compared techniques for mixing and placing MTA and concluded that mixing with ultrasonic methods and placing it manually results in minimal voids within the MTA [18]. In our current case report, the tissue at the perforation site was repaired using MTA that was mixed and placed manually, and minor porosity was observed in Tooth Number 33 on the periapical radiograph. However, this did not affect the success of the treatment. MTA requires moisture for its setting mechanism to occur. In the treated teeth, MTA was left in place with wet cotton to allow the setting mechanism to complete.

The bioactive properties of MTA play an important role in the success of treatment [19]. The ability of calcium silicate–based cements to form hydroxyapatite supports tissue regeneration [20]. However, it should be noted that MTA is osteoconductive, meaning it supports bone formation on its surface rather than directly inducing it. Therefore, the osteoconductive properties of MTA and its ability to form hydroxyapatite have enhanced the success of the treatment by supporting bone regeneration in our current case.

The time elapsed between the iatrogenic event and repair is a critical determinant of prognosis. Perforations repaired immediately or within a short timeframe generally exhibit higher success rates due to minimal microbial colonization and inflammatory response [5]. However, in our current case, approximately 1 year had passed since the initial RCT attempt where the perforations likely occurred. While this delay introduced a risk of established infection (evidenced by apical radiolucencies and the diagnosis of asymptomatic apical periodontitis), the effective chemomechanical debridement, extended calcium hydroxide dressing (14 days), and subsequent MTA seal successfully mitigated this risk, leading to periapical healing. This underscores the importance of thorough disinfection protocols, particularly in delayed repair scenarios.

The prognosis of perforation repair depends on perforation size, location, time of repair, microbial contamination, and the sealing material [21]. The relatively small size of both perforations (< 1 mm) in this case, significantly below the 3 mm threshold associated with compromised prognosis [11], was a favorable prognostic factor contributing to the successful seal achieved with MTA. This aligns with clinical evidence indicating superior outcomes in smaller perforations. Specifically, Siew et al. [10] demonstrated in their meta-analysis that perforations < 1 mm had an 89% success rate versus 42% in > 3 mm cases, while Mente et al. [11] reported a 92% tooth survival at 10-year follow-up for sub-1 mm perforations.

The literature states that large perforations (greater than 3 mm) and those close to the alveolar crest (within 2 mm) reduce the chances of success [11]. In the current case, the small size of the perforations and their distance from the alveolar crest being over 5 mm provided an advantage for a positive prognosis. This finding is strongly supported by the seminal work of Fuss and Trope [22], who concluded that the location of the perforation is probably the most important factor affecting the prognosis, with crestal root perforations being the most susceptible to epithelial migration and rapid pocket formation, thus having the lowest repair success rates.

The limitations of the nonsurgical approach should be considered. In cases of apical perforations or when orthograde access is not possible, surgical intervention may be necessary [23]. The current case contributes to the literature by demonstrating the successful management of simultaneous double perforations in Teeth 33 and 34 using MTA guided by CBCT while also highlighting the importance of meticulous planning in calcified canals. However, it should be noted that this approach may not be suitable in every case. For instance, in situations involving significant periodontal damage, untreatable infection, or when restoration is not possible, alternative approaches such as surgical repair or tooth extraction should be considered.

## 4. Conclusion

This case demonstrates the successful nonsurgical management of simultaneous double lateral root perforations in adjacent mandibular teeth using MTA, critically guided by CBCT. The millimetric localization and angular measurements provided by CBCT were indispensable for planning a precise, nontraumatic access path to locate the unprepared canals, a key factor in achieving a favorable outcome. This approach highlights the value of CBCT beyond diagnosis into active treatment planning for complex iatrogenic errors.

## Data Availability

The data that supports the findings of this study are available from the corresponding author upon reasonable request.

## Patient Perspective

The patient expressed satisfaction with the treatment process and outcomes, stating, “I'm so glad you are here; thanks to you, my teeth have healed, I have no pain, and I can use them.”
